# Supplementary material for: Identification of photoperiod-induced specific miRNAs in the adrenal glands of Sunite sheep (Ovis aries)
Source: Front Vet Sci. 2022 Jul 22;9:888207. doi: 10.3389/fvets.2022.888207 (PMC9354845; doi:10.3389/fvets.2022.888207)
Supplement: Supplementary file 1 [file Data_Sheet_1.ZIP › Supplementary file/miRDeep2 file.zip!/miRDeep2 file/Know_Structure/LP42A3/oar-mir-362.pdf]

miRBase precursor : oar-mir-362
Total read count : 1508
oar-miR-362 read count : 1494
remaining reads : 14

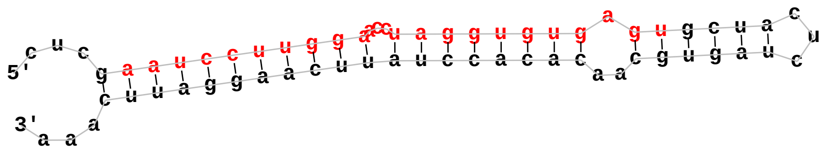

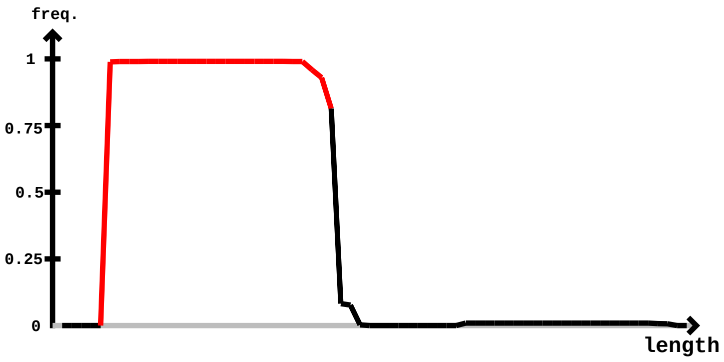


```
       oar-miR-362
5'- cucgaauccuuggaaccuaggugugagugcuacucuagugcaacacaccuauucaaggauucaaa   -3'    exp
    ...(((((((((((...((((((((.((((((...))))))..)))))))))))))))))))...    reads      mm         sample
    ....aauccuuggaaccuaggug..........................................    1          0          seq
    ....aauccuuggaaccuaggugug........................................    47         0          seq
    ....aauccuuggaaccuagguguga.......................................    44         0          seq
    ....aauccuuggaaccuaggugugag......................................    175        0          seq
    ....aauccuuggaaccuaggugugagu.....................................    1101       0          seq
    ....aauccuuggaaccuaggugugagug....................................    8          0          seq
    ....aauccuuggaaccuaggugugagugc...................................    111        0          seq
    ....aauccuuggaaccuaggugugagugcu..................................    4          0          seq
    .....auccuuggaaccuaggugugagu.....................................    1          0          seq
    .....auccuuggaaccuaggugugagugc...................................    1          0          seq
    ........cuuggaaccuaggugugagu.....................................    1          0          seq
    .........................................aacacaccuauucaaggauu....    3          0          seq
    .........................................aacacaccuauucaaggauuc...    1          0          seq
    .........................................aacacaccuauucaaggauuca..    10         0          seq
```
